# Bis[2-(2-pyridylmethyl­eneamino)benzene­sulfonato]-κ^3^ *N*,*N*′,*O*;κ^2^ *N*,*N*′-copper(II)

**DOI:** 10.1107/S1600536809035661

**Published:** 2009-09-12

**Authors:** Ge-Ge Yang, Miao Ou-Yang, Xiu-Jin Meng, Xue-Ren Huang, Yi-Min Jiang

**Affiliations:** a College of Chemistry and Chemical Engineering, Guangxi Normal University, Guilin, Guangxi 541004, People’s Republic of China

## Abstract

In the mononuclear title compound, [Cu(C_12_H_9_N_2_O_3_S)_2_],  the copper(II) salt of 2-(2-pyridylmethyl­eneamino)benzene­sulfonic acid, the Cu^II^ atom is coordinated by one O and two N atoms from a monoanion as well as by two N atoms from another monoanion in a distorted trigonal-bipyramidal environment.

## Related literature

For the synthesis of the ligand, see: Casella & Gullotti (1986 ▶). For the structures of analogues, see: Cai *et al.* (2008 ▶). For related Schiff base complexes, see: Li *et al.* (2006 ▶, 2007 ▶); Wang *et al.* (1994 ▶); Jiang *et al.* (2006 ▶); Zhang *et al.* (2004 ▶). For a discussion on self-assembly, see: Zheng *et al.* (2001 ▶).
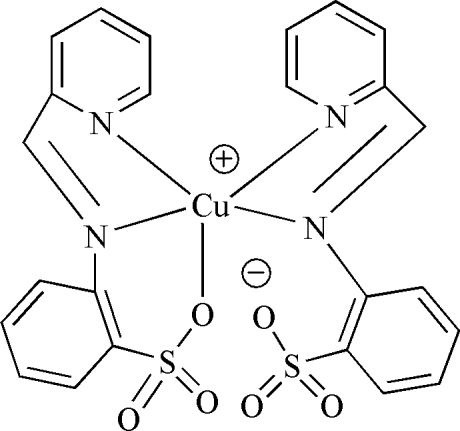


## Experimental

### 

#### Crystal data

[Cu(C_12_H_9_N_2_O_3_S)_2_]
*M* *_r_* = 586.08
Orthorhombic, 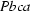

*a* = 17.347 (4) Å
*b* = 14.686 (4) Å
*c* = 18.830 (5) Å
*V* = 4797 (2) Å^3^
*Z* = 8
Mo *K*α radiation
μ = 1.14 mm^−1^
*T* = 294 K
0.25 × 0.19 × 0.13 mm

#### Data collection

Bruker SMART CCD area-detector diffractometer
Absorption correction: multi-scan (*SADABS*; Sheldrick, 1996 ▶) *T*_min_ = 0.762, *T*_max_ = 0.868
33042 measured reflections
4455 independent reflections
2420 reflections with *I* > 2σ(*I*)
*R*_int_ = 0.128

#### Refinement

*R*[*F* ^2^ > 2σ(*F* ^2^)] = 0.052
*wR*(*F* ^2^) = 0.131
*S* = 1.01
4455 reflections
334 parameters
H-atom parameters constrained
Δρ_max_ = 0.42 e Å^−3^
Δρ_min_ = −0.64 e Å^−3^

### 

Data collection: *SMART* (Bruker, 2004 ▶); cell refinement: *SAINT* (Bruker, 2004 ▶); data reduction: *SAINT*; program(s) used to solve structure: *SHELXS97* (Sheldrick, 2008 ▶); program(s) used to refine structure: *SHELXL97* (Sheldrick, 2008 ▶); molecular graphics: *SHELXTL* (Sheldrick, 2008 ▶); software used to prepare material for publication: *SHELXTL*.

## Supplementary Material

Crystal structure: contains datablocks I, global. DOI: 10.1107/S1600536809035661/ng2628sup1.cif

Structure factors: contains datablocks I. DOI: 10.1107/S1600536809035661/ng2628Isup2.hkl

Additional supplementary materials:  crystallographic information; 3D view; checkCIF report

## Figures and Tables

**Fig. 1.**
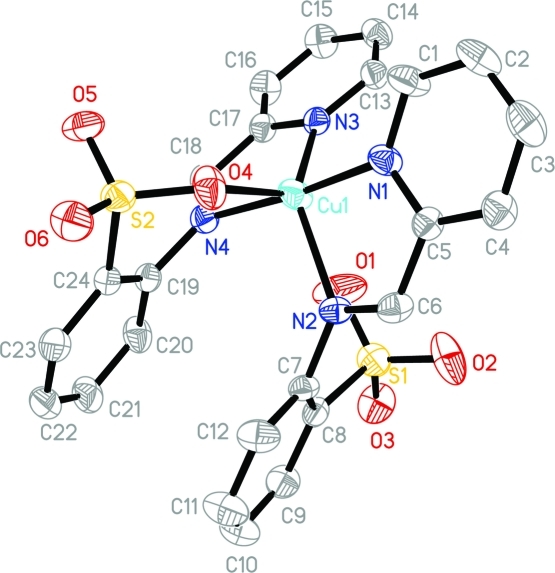
The molecular structure of the title complex, An ellipsoid plot (30% probability) showing the numbering scheme.

**Fig. 2.**
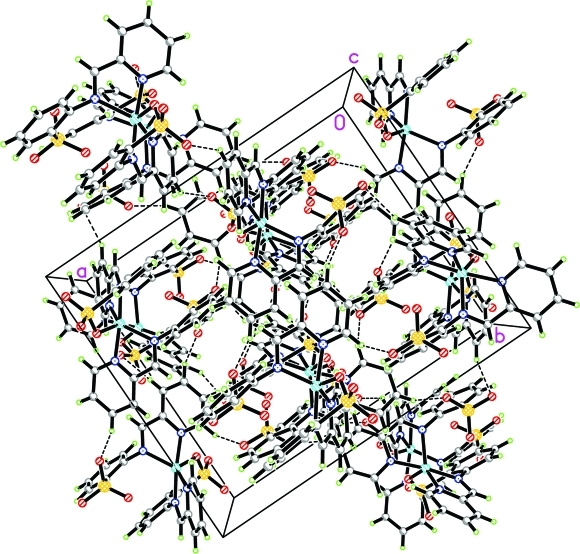
Packing of the title complex, showing the form a there-dimensional superamolecular structure, linked via atypical hydrogen bonds(dashed lines).

## supplementary crystallographic information

### Comment

Nowadays, Great interesting have focused on the design and control of the network superamolecular coordination complexes. Via utilizing both coordination bonds and hydrogen bonds in the self-assembly chemistry (Zheng *et al.*, 2001). However, Schiff base complexes which contain both sulfur and amino acid functionalities have received much attention owing to their potential application in medicine (Casella & Gullotti, 1986; Wang *et al.*, 1994; Li *et al.*, 2006).

Our group have focused on the exploration of the coordination chemistry of the sulfonate ligand for many years. (Zhang *et al.*, 2004; Jiang *et al.*, 2006; Li *et al.*,2007). In this work, we report the synthesis and the stucture of the mononuclear Cu^II^ complex(Fig. 1). The unit of structure is composed of one Cu^II^, two deprotonated Paba^-^ ligands. The five-coordinated Cu^II^ atom has a distorted distorted trigonal biyramid geometry, being coordinated by pyridine N, imine N and sulfonate O atoms from one of the deprotonated Paba^-^ligands in a tridentate facial arrangement. And the other pyridine N and imine N from another deprotonated Paba^-^ ligands in a bidentate facial arrangement. It is notable that the sulfonate O atom doesn't participate in coordinating, Which is different from those reported complexes with N,*N*',*O*-tridentate donor ligands (Cai *et al.*, 2008).

The point deserves mention that there exits many atypical hydrogen bonds. In which the C—H donor and the S—O acceptor group of the Paba ligands participate in the hydrogen bonding and form a three-dimensional superamolecular structure(Fig.2).

### Experimental

The potassium salt of 2-(2-pyridylmethylimine)benzenesulfonic acid (PabaK) was synthesized according to the literature methods (Casella *et al.*, 1986).

For the preparation of the title complex, the ligand pabaK (1 mmol, 0.30 g) was dissolved in methanol (10 ml) at 333 K and an aqueous solution (10 ml) containing 0.90 g Cu(AcO)_2_.H_2_O (0.5 mmol,0.90 g) was added to the above solution. The resulting solution was stirred at 333 K for 4 h, then cooled to room temperature and filtrated. A blue-block crystal suitable for X-ray diffraction were obtained by slow evaporation after several days in a yield of 55%. Analysis found for (%.): C: 49.14, H: 3.07,*N*: 9.56,*S*: 10.92; C_24_H_18_CuN_4_O_6_S_2_ requires (%.): C: 49.09, H: 3.09, N: 9.53, S: 10.95.

### Refinement

H atoms bonded to C were positioned geometrically with C—H distance 0.93 Å, and treated as riding atoms,with *U*_iso_(H)= 1.2*U*_eq_(C).

### Crystal data

**Table:**

[Cu(C_12_H_9_N_2_O_3_S)_2_]	*D*_x_ = 1.623 Mg m^−^^3^
*M**_r_* = 586.08	Mo *K*α radiation, λ = 0.71073 Å
Orthorhombic, *P**b**c**a*	Cell parameters from 1929 reflections
*a* = 17.347 (4) Å	θ = 2.4–18.1°
*b* = 14.686 (4) Å	µ = 1.14 mm^−^^1^
*c* = 18.830 (5) Å	*T* = 294 K
*V* = 4797 (2) Å^3^	Block, blue
*Z* = 8	0.25 × 0.19 × 0.13 mm
*F*(000) = 2392	

### Data collection

**Table:**

Bruker SMART CCD area-detector diffractometer	4455 independent reflections
Radiation source: fine-focus sealed tube	2420 reflections with *I* > 2σ(*I*)
graphite	*R*_int_ = 0.128
φ and ω scans	θ_max_ = 25.5°, θ_min_ = 2.4°
Absorption correction: multi-scan (*SADABS*; Sheldrick, 1996)	*h* = −20→21
*T*_min_ = 0.762, *T*_max_ = 0.868	*k* = −17→17
33042 measured reflections	*l* = −22→22

### Refinement

**Table:**

Refinement on *F*^2^	Primary atom site location: structure-invariant direct methods
Least-squares matrix: full	Secondary atom site location: difference Fourier map
*R*[*F*^2^ > 2σ(*F*^2^)] = 0.052	Hydrogen site location: inferred from neighbouring sites
*wR*(*F*^2^) = 0.131	H-atom parameters constrained
*S* = 1.01	*w* = 1/[σ^2^(*F*_o_^2^) + (0.0346*P*)^2^ + 8.08*P*] where *P* = (*F*_o_^2^ + 2*F*_c_^2^)/3
4455 reflections	(Δ/σ)_max_ < 0.001
334 parameters	Δρ_max_ = 0.42 e Å^−^^3^
0 restraints	Δρ_min_ = −0.64 e Å^−^^3^

### Special details

**Table:**

Geometry. All e.s.d.'s (except the e.s.d. in the dihedral angle between two l.s. planes)are estimated using the full covariance matrix. The cell e.s.d.'s are takeninto account individually in the estimation of e.s.d.'s in distances, anglesand torsion angles; correlations between e.s.d.'s in cell parameters are onlyused when they are defined by crystal symmetry. An approximate (isotropic)treatment of cell e.s.d.'s is used for estimating e.s.d.'s involving l.s. planes.
Refinement. Refinement of *F*^2^ against ALL reflections. The weighted *R*-factor *wR* and goodness of fit *S* are based on *F*^2^, conventional *R*-factors *R* are based on *F*, with *F* set to zero for negative *F*^2^. The threshold expression of *F*^2^ > σ(*F*^2^) is used only for calculating *R*-factors(gt) *etc*. and is not relevant to the choice of reflections for refinement. *R*-factors based on *F*^2^ are statistically about twice as large as those based on *F*, and *R*- factors based on ALL data will be even larger.

### Fractional atomic coordinates and isotropic or equivalent isotropic displacement parameters (Å^2^)

**Table:**

	*x*	*y*	*z*	*U*_iso_*/*U*_eq_	
Cu1	0.06065 (4)	0.76356 (4)	0.39106 (4)	0.0524 (2)	
S1	0.26315 (8)	0.67187 (12)	0.44099 (8)	0.0587 (4)	
S2	0.01410 (8)	0.84439 (10)	0.23757 (8)	0.0508 (4)	
O1	0.2221 (3)	0.7519 (4)	0.4561 (2)	0.1110 (19)	
O2	0.2297 (2)	0.5889 (3)	0.4688 (2)	0.0936 (16)	
O3	0.3437 (2)	0.6766 (3)	0.4590 (2)	0.0734 (12)	
O4	−0.00046 (19)	0.7751 (3)	0.2916 (2)	0.0664 (11)	
O5	−0.0112 (2)	0.9335 (3)	0.2599 (2)	0.0657 (11)	
O6	−0.0135 (2)	0.8176 (3)	0.16862 (19)	0.0657 (11)	
N1	−0.0138 (2)	0.6688 (3)	0.4184 (2)	0.0470 (11)	
N2	0.1189 (2)	0.6475 (3)	0.3528 (2)	0.0389 (10)	
N3	0.0584 (2)	0.8399 (3)	0.4808 (2)	0.0417 (10)	
N4	0.1258 (2)	0.8652 (3)	0.3585 (2)	0.0426 (10)	
C1	−0.0816 (3)	0.6815 (4)	0.4503 (3)	0.0638 (17)	
H1	−0.0966	0.7406	0.4614	0.077*	
C2	−0.1304 (3)	0.6107 (5)	0.4675 (3)	0.0655 (17)	
H2	−0.1770	0.6218	0.4905	0.079*	
C3	−0.1092 (3)	0.5243 (5)	0.4501 (3)	0.0678 (18)	
H3	−0.1410	0.4754	0.4614	0.081*	
C4	−0.0397 (3)	0.5095 (4)	0.4155 (3)	0.0543 (15)	
H4	−0.0243	0.4510	0.4030	0.065*	
C5	0.0056 (3)	0.5828 (3)	0.4000 (3)	0.0428 (13)	
C6	0.0795 (3)	0.5753 (4)	0.3622 (3)	0.0465 (13)	
H6	0.0969	0.5193	0.3455	0.056*	
C7	0.1900 (3)	0.6409 (3)	0.3139 (3)	0.0392 (12)	
C8	0.2598 (3)	0.6566 (3)	0.3484 (3)	0.0429 (13)	
C9	0.3270 (3)	0.6531 (4)	0.3078 (3)	0.0574 (16)	
H9	0.3741	0.6640	0.3296	0.069*	
C10	0.3253 (4)	0.6338 (4)	0.2365 (4)	0.077 (2)	
H10	0.3708	0.6312	0.2106	0.092*	
C11	0.2559 (4)	0.6184 (5)	0.2040 (4)	0.0780 (19)	
H11	0.2543	0.6056	0.1557	0.094*	
C12	0.1886 (3)	0.6219 (4)	0.2425 (3)	0.0608 (16)	
H12	0.1418	0.6112	0.2199	0.073*	
C13	0.0264 (3)	0.8255 (4)	0.5440 (3)	0.0465 (13)	
H13	−0.0012	0.7719	0.5508	0.056*	
C14	0.0320 (3)	0.8859 (4)	0.6002 (3)	0.0538 (15)	
H14	0.0086	0.8727	0.6434	0.065*	
C15	0.0722 (3)	0.9648 (4)	0.5915 (3)	0.0595 (16)	
H15	0.0753	1.0073	0.6280	0.071*	
C16	0.1081 (3)	0.9804 (4)	0.5275 (3)	0.0584 (15)	
H16	0.1370	1.0329	0.5206	0.070*	
C17	0.1007 (3)	0.9174 (3)	0.4737 (3)	0.0445 (13)	
C18	0.1366 (3)	0.9272 (3)	0.4042 (3)	0.0467 (13)	
H18	0.1667	0.9778	0.3937	0.056*	
C19	0.1617 (3)	0.8674 (3)	0.2902 (3)	0.0420 (13)	
C20	0.2410 (3)	0.8767 (4)	0.2847 (3)	0.0550 (15)	
H20	0.2707	0.8865	0.3251	0.066*	
C21	0.2755 (3)	0.8713 (4)	0.2187 (3)	0.0656 (17)	
H21	0.3286	0.8784	0.2147	0.079*	
C22	0.2316 (3)	0.8556 (4)	0.1586 (3)	0.0640 (17)	
H22	0.2550	0.8516	0.1143	0.077*	
C23	0.1524 (3)	0.8457 (4)	0.1651 (3)	0.0544 (15)	
H23	0.1229	0.8348	0.1248	0.065*	
C24	0.1167 (3)	0.8516 (3)	0.2304 (3)	0.0412 (12)	

### Atomic displacement parameters (Å^2^)

**Table:**

	*U*^11^	*U*^22^	*U*^33^	*U*^12^	*U*^13^	*U*^23^
Cu1	0.0531 (4)	0.0415 (4)	0.0625 (5)	−0.0138 (3)	0.0194 (4)	−0.0088 (3)
S1	0.0445 (9)	0.0764 (11)	0.0553 (10)	0.0017 (8)	−0.0076 (7)	0.0064 (8)
S2	0.0407 (8)	0.0617 (10)	0.0498 (9)	0.0054 (7)	−0.0020 (7)	0.0020 (8)
O1	0.120 (4)	0.153 (5)	0.060 (3)	0.080 (4)	−0.033 (3)	−0.043 (3)
O2	0.077 (3)	0.132 (4)	0.071 (3)	−0.044 (3)	−0.009 (2)	0.036 (3)
O3	0.053 (2)	0.078 (3)	0.089 (3)	−0.008 (2)	−0.029 (2)	0.003 (2)
O4	0.045 (2)	0.086 (3)	0.068 (3)	−0.014 (2)	−0.0058 (19)	0.028 (2)
O5	0.054 (2)	0.069 (3)	0.074 (3)	0.021 (2)	0.004 (2)	−0.009 (2)
O6	0.057 (2)	0.085 (3)	0.055 (3)	0.001 (2)	−0.008 (2)	−0.007 (2)
N1	0.039 (3)	0.047 (3)	0.055 (3)	−0.008 (2)	0.009 (2)	−0.004 (2)
N2	0.030 (2)	0.041 (3)	0.046 (3)	−0.002 (2)	−0.0006 (19)	−0.008 (2)
N3	0.040 (2)	0.036 (2)	0.050 (3)	−0.001 (2)	0.003 (2)	0.004 (2)
N4	0.042 (3)	0.042 (3)	0.044 (3)	−0.001 (2)	0.001 (2)	0.001 (2)
C1	0.048 (4)	0.066 (4)	0.077 (4)	−0.010 (3)	0.024 (3)	−0.017 (3)
C2	0.049 (4)	0.089 (5)	0.059 (4)	−0.019 (3)	0.017 (3)	−0.014 (4)
C3	0.063 (4)	0.074 (5)	0.067 (4)	−0.034 (4)	0.007 (3)	−0.002 (4)
C4	0.056 (4)	0.045 (3)	0.062 (4)	−0.013 (3)	0.000 (3)	−0.004 (3)
C5	0.041 (3)	0.039 (3)	0.048 (3)	−0.005 (2)	−0.004 (3)	−0.001 (3)
C6	0.041 (3)	0.039 (3)	0.059 (4)	−0.002 (3)	−0.002 (3)	−0.012 (3)
C7	0.032 (3)	0.038 (3)	0.048 (3)	0.003 (2)	0.003 (2)	−0.003 (2)
C8	0.036 (3)	0.037 (3)	0.056 (3)	0.001 (2)	−0.002 (3)	0.009 (3)
C9	0.030 (3)	0.061 (4)	0.081 (5)	−0.004 (3)	0.004 (3)	0.010 (3)
C10	0.061 (5)	0.090 (5)	0.079 (5)	0.012 (4)	0.028 (4)	0.003 (4)
C11	0.069 (5)	0.100 (5)	0.064 (4)	0.007 (4)	0.015 (4)	−0.015 (4)
C12	0.049 (4)	0.073 (4)	0.061 (4)	0.003 (3)	−0.002 (3)	−0.018 (3)
C13	0.043 (3)	0.048 (3)	0.049 (4)	−0.003 (3)	0.003 (3)	0.008 (3)
C14	0.048 (3)	0.072 (4)	0.042 (4)	−0.003 (3)	0.000 (3)	0.003 (3)
C15	0.069 (4)	0.066 (4)	0.044 (4)	−0.005 (3)	−0.009 (3)	−0.010 (3)
C16	0.073 (4)	0.050 (4)	0.052 (4)	−0.008 (3)	−0.009 (3)	−0.007 (3)
C17	0.045 (3)	0.045 (3)	0.043 (3)	0.000 (3)	−0.003 (3)	0.004 (3)
C18	0.052 (3)	0.039 (3)	0.049 (4)	−0.014 (3)	−0.002 (3)	0.004 (3)
C19	0.046 (3)	0.034 (3)	0.047 (3)	−0.001 (2)	0.004 (3)	0.005 (2)
C20	0.047 (4)	0.062 (4)	0.056 (4)	−0.015 (3)	−0.002 (3)	0.015 (3)
C21	0.047 (4)	0.078 (5)	0.071 (5)	−0.005 (3)	0.012 (3)	0.025 (4)
C22	0.057 (4)	0.082 (5)	0.054 (4)	0.014 (3)	0.014 (3)	0.015 (3)
C23	0.056 (4)	0.061 (4)	0.046 (4)	0.009 (3)	−0.004 (3)	0.005 (3)
C24	0.042 (3)	0.040 (3)	0.041 (3)	0.003 (2)	0.003 (3)	0.006 (2)

### Geometric parameters (Å, °)

**Table:**

Cu1—N1	1.967 (4)	C7—C12	1.374 (7)
Cu1—N4	1.970 (4)	C7—C8	1.394 (6)
Cu1—N3	2.028 (4)	C8—C9	1.394 (7)
Cu1—N2	2.108 (4)	C9—C10	1.372 (8)
Cu1—O4	2.158 (4)	C9—H9	0.9300
S1—O1	1.403 (4)	C10—C11	1.368 (8)
S1—O3	1.439 (4)	C10—H10	0.9300
S1—O2	1.447 (4)	C11—C12	1.374 (8)
S1—C8	1.758 (5)	C11—H11	0.9300
S2—O6	1.439 (4)	C12—H12	0.9300
S2—O5	1.443 (4)	C13—C14	1.384 (7)
S2—O4	1.462 (4)	C13—H13	0.9300
S2—C24	1.788 (5)	C14—C15	1.364 (7)
N1—C1	1.334 (6)	C14—H14	0.9300
N1—C5	1.352 (6)	C15—C16	1.375 (7)
N2—C6	1.275 (6)	C15—H15	0.9300
N2—C7	1.438 (6)	C16—C17	1.379 (7)
N3—C13	1.329 (6)	C16—H16	0.9300
N3—C17	1.361 (6)	C17—C18	1.456 (7)
N4—C18	1.267 (6)	C18—H18	0.9300
N4—C19	1.430 (6)	C19—C20	1.387 (7)
C1—C2	1.379 (7)	C19—C24	1.388 (7)
C1—H1	0.9300	C20—C21	1.380 (7)
C2—C3	1.362 (8)	C20—H20	0.9300
C2—H2	0.9300	C21—C22	1.383 (8)
C3—C4	1.388 (7)	C21—H21	0.9300
C3—H3	0.9300	C22—C23	1.387 (7)
C4—C5	1.364 (7)	C22—H22	0.9300
C4—H4	0.9300	C23—C24	1.379 (7)
C5—C6	1.470 (7)	C23—H23	0.9300
C6—H6	0.9300		
			
N1—Cu1—N4	173.80 (17)	C12—C7—N2	119.8 (5)
N1—Cu1—N3	99.20 (17)	C8—C7—N2	119.7 (4)
N4—Cu1—N3	81.47 (17)	C9—C8—C7	117.6 (5)
N1—Cu1—N2	80.34 (16)	C9—C8—S1	121.4 (4)
N4—Cu1—N2	103.36 (16)	C7—C8—S1	120.8 (4)
N3—Cu1—N2	137.83 (15)	C10—C9—C8	121.8 (6)
N1—Cu1—O4	87.73 (15)	C10—C9—H9	119.1
N4—Cu1—O4	87.30 (16)	C8—C9—H9	119.1
N3—Cu1—O4	132.05 (16)	C11—C10—C9	119.3 (6)
N2—Cu1—O4	90.12 (15)	C11—C10—H10	120.3
O1—S1—O3	113.8 (3)	C9—C10—H10	120.3
O1—S1—O2	115.3 (3)	C10—C11—C12	120.4 (6)
O3—S1—O2	110.1 (3)	C10—C11—H11	119.8
O1—S1—C8	107.0 (2)	C12—C11—H11	119.8
O3—S1—C8	105.8 (3)	C7—C12—C11	120.5 (6)
O2—S1—C8	103.8 (3)	C7—C12—H12	119.7
O6—S2—O5	114.2 (2)	C11—C12—H12	119.7
O6—S2—O4	112.4 (2)	N3—C13—C14	123.6 (5)
O5—S2—O4	112.1 (2)	N3—C13—H13	118.2
O6—S2—C24	106.2 (2)	C14—C13—H13	118.2
O5—S2—C24	105.7 (2)	C15—C14—C13	119.3 (5)
O4—S2—C24	105.4 (2)	C15—C14—H14	120.4
S2—O4—Cu1	125.0 (2)	C13—C14—H14	120.4
C1—N1—C5	117.7 (5)	C14—C15—C16	118.5 (5)
C1—N1—Cu1	126.7 (4)	C14—C15—H15	120.7
C5—N1—Cu1	115.5 (3)	C16—C15—H15	120.7
C6—N2—C7	118.3 (4)	C15—C16—C17	119.4 (5)
C6—N2—Cu1	111.6 (3)	C15—C16—H16	120.3
C7—N2—Cu1	129.8 (3)	C17—C16—H16	120.3
C13—N3—C17	116.5 (4)	N3—C17—C16	122.6 (5)
C13—N3—Cu1	131.7 (3)	N3—C17—C18	113.7 (5)
C17—N3—Cu1	111.7 (3)	C16—C17—C18	123.6 (5)
C18—N4—C19	122.1 (4)	N4—C18—C17	118.4 (5)
C18—N4—Cu1	114.7 (3)	N4—C18—H18	120.8
C19—N4—Cu1	123.1 (3)	C17—C18—H18	120.8
N1—C1—C2	122.9 (6)	C20—C19—C24	120.9 (5)
N1—C1—H1	118.6	C20—C19—N4	120.0 (5)
C2—C1—H1	118.6	C24—C19—N4	118.8 (4)
C3—C2—C1	118.6 (6)	C21—C20—C19	119.4 (5)
C3—C2—H2	120.7	C21—C20—H20	120.3
C1—C2—H2	120.7	C19—C20—H20	120.3
C2—C3—C4	119.6 (5)	C20—C21—C22	120.5 (5)
C2—C3—H3	120.2	C20—C21—H21	119.8
C4—C3—H3	120.2	C22—C21—H21	119.8
C5—C4—C3	118.5 (5)	C21—C22—C23	119.4 (5)
C5—C4—H4	120.7	C21—C22—H22	120.3
C3—C4—H4	120.7	C23—C22—H22	120.3
N1—C5—C4	122.6 (5)	C24—C23—C22	121.1 (5)
N1—C5—C6	114.3 (4)	C24—C23—H23	119.4
C4—C5—C6	123.1 (5)	C22—C23—H23	119.4
N2—C6—C5	118.1 (5)	C23—C24—C19	118.7 (5)
N2—C6—H6	120.9	C23—C24—S2	120.7 (4)
C5—C6—H6	120.9	C19—C24—S2	120.5 (4)
C12—C7—C8	120.4 (5)		
			
O6—S2—O4—Cu1	158.9 (3)	C6—N2—C7—C8	−113.4 (5)
O5—S2—O4—Cu1	−70.9 (3)	Cu1—N2—C7—C8	73.1 (6)
C24—S2—O4—Cu1	43.6 (3)	C12—C7—C8—C9	0.6 (7)
N1—Cu1—O4—S2	172.6 (3)	N2—C7—C8—C9	−177.5 (4)
N4—Cu1—O4—S2	−3.7 (3)	C12—C7—C8—S1	−174.8 (4)
N3—Cu1—O4—S2	72.2 (3)	N2—C7—C8—S1	7.2 (6)
N2—Cu1—O4—S2	−107.1 (3)	O1—S1—C8—C9	120.6 (5)
N4—Cu1—N1—C1	−51.2 (18)	O3—S1—C8—C9	−1.1 (5)
N3—Cu1—N1—C1	44.4 (5)	O2—S1—C8—C9	−117.1 (4)
N2—Cu1—N1—C1	−178.3 (5)	O1—S1—C8—C7	−64.3 (5)
O4—Cu1—N1—C1	−87.8 (5)	O3—S1—C8—C7	174.1 (4)
N4—Cu1—N1—C5	126.0 (16)	O2—S1—C8—C7	58.1 (5)
N3—Cu1—N1—C5	−138.4 (4)	C7—C8—C9—C10	−0.8 (8)
N2—Cu1—N1—C5	−1.1 (4)	S1—C8—C9—C10	174.6 (5)
O4—Cu1—N1—C5	89.4 (4)	C8—C9—C10—C11	0.6 (10)
N1—Cu1—N2—C6	2.5 (3)	C9—C10—C11—C12	−0.3 (10)
N4—Cu1—N2—C6	−172.4 (3)	C8—C7—C12—C11	−0.3 (8)
N3—Cu1—N2—C6	95.6 (4)	N2—C7—C12—C11	177.8 (5)
O4—Cu1—N2—C6	−85.1 (4)	C10—C11—C12—C7	0.1 (10)
N1—Cu1—N2—C7	176.4 (4)	C17—N3—C13—C14	2.4 (7)
N4—Cu1—N2—C7	1.5 (4)	Cu1—N3—C13—C14	179.0 (4)
N3—Cu1—N2—C7	−90.5 (4)	N3—C13—C14—C15	0.0 (8)
O4—Cu1—N2—C7	88.8 (4)	C13—C14—C15—C16	−2.1 (8)
N1—Cu1—N3—C13	9.2 (5)	C14—C15—C16—C17	1.7 (8)
N4—Cu1—N3—C13	−177.1 (5)	C13—N3—C17—C16	−2.7 (7)
N2—Cu1—N3—C13	−76.6 (5)	Cu1—N3—C17—C16	−179.9 (4)
O4—Cu1—N3—C13	104.4 (4)	C13—N3—C17—C18	177.6 (4)
N1—Cu1—N3—C17	−174.1 (3)	Cu1—N3—C17—C18	0.4 (5)
N4—Cu1—N3—C17	−0.4 (3)	C15—C16—C17—N3	0.7 (8)
N2—Cu1—N3—C17	100.2 (4)	C15—C16—C17—C18	−179.7 (5)
O4—Cu1—N3—C17	−78.8 (4)	C19—N4—C18—C17	−177.2 (4)
N1—Cu1—N4—C18	96.9 (17)	Cu1—N4—C18—C17	−0.2 (6)
N3—Cu1—N4—C18	0.3 (4)	N3—C17—C18—N4	−0.1 (7)
N2—Cu1—N4—C18	−137.0 (4)	C16—C17—C18—N4	−179.8 (5)
O4—Cu1—N4—C18	133.5 (4)	C18—N4—C19—C20	53.8 (7)
N1—Cu1—N4—C19	−86.1 (17)	Cu1—N4—C19—C20	−122.9 (4)
N3—Cu1—N4—C19	177.3 (4)	C18—N4—C19—C24	−132.0 (5)
N2—Cu1—N4—C19	40.0 (4)	Cu1—N4—C19—C24	51.2 (6)
O4—Cu1—N4—C19	−49.5 (4)	C24—C19—C20—C21	0.8 (8)
C5—N1—C1—C2	2.7 (8)	N4—C19—C20—C21	174.8 (5)
Cu1—N1—C1—C2	179.9 (4)	C19—C20—C21—C22	−0.9 (9)
N1—C1—C2—C3	−1.2 (9)	C20—C21—C22—C23	0.4 (9)
C1—C2—C3—C4	−0.4 (9)	C21—C22—C23—C24	0.2 (9)
C2—C3—C4—C5	0.3 (9)	C22—C23—C24—C19	−0.3 (8)
C1—N1—C5—C4	−2.8 (8)	C22—C23—C24—S2	176.9 (4)
Cu1—N1—C5—C4	179.7 (4)	C20—C19—C24—C23	−0.2 (8)
C1—N1—C5—C6	177.2 (5)	N4—C19—C24—C23	−174.3 (4)
Cu1—N1—C5—C6	−0.3 (6)	C20—C19—C24—S2	−177.4 (4)
C3—C4—C5—N1	1.4 (8)	N4—C19—C24—S2	8.5 (6)
C3—C4—C5—C6	−178.6 (5)	O6—S2—C24—C23	9.3 (5)
C7—N2—C6—C5	−178.1 (4)	O5—S2—C24—C23	−112.4 (4)
Cu1—N2—C6—C5	−3.5 (6)	O4—S2—C24—C23	128.7 (4)
N1—C5—C6—N2	2.6 (7)	O6—S2—C24—C19	−173.6 (4)
C4—C5—C6—N2	−177.3 (5)	O5—S2—C24—C19	64.7 (5)
C6—N2—C7—C12	68.5 (6)	O4—S2—C24—C19	−54.1 (5)
Cu1—N2—C7—C12	−105.0 (5)		
